# Association between Cigarette Smoking Status and Composition of Gut Microbiota: Population-Based Cross-Sectional Study

**DOI:** 10.3390/jcm7090282

**Published:** 2018-09-14

**Authors:** Su Hwan Lee, Yeojun Yun, Soo Jung Kim, Eun-Ju Lee, Yoosoo Chang, Seungho Ryu, Hocheol Shin, Hyung-Lae Kim, Han-Na Kim, Jin Hwa Lee

**Affiliations:** 1 Division of Pulmonary and Critical Care Medicine, Department of Internal Medicine, College of Medicine, Ewha Womans University, 1071 Anyangcheon-ro, Yangcheon-gu, Seoul 07985, Korea; hihogogo@naver.com (S.H.L.); crystalkim38@gmail.com (S.J.K.); 2 Department of Biochemistry, College of Medicine, Ewha Womans University, Seoul 07985, Korea; yeojuny@gmail.com (Y.Y.); ionantha97@gmail.com (E.-J.L.); hyung@ewha.ac.kr (H.-L.K.); 3 Center for Cohort Studies, Total Healthcare Center, Kangbuk Samsung Hospital, Sungkyunkwan University School of Medicine, Seoul 03181, Korea; yoosoo.chang@samsung.com (Y.C.); sh703.yoo@samsung.com (S.R.); 4 Department of Occupational and Environmental Medicine, Kangbuk Samsung Hospital, Sungkyunkwan University, School of Medicine, Seoul 03181, Korea; 5 Department of Family Medicine, Kangbuk Samsung Hospital, Sungkyunkwan University School of Medicine, Seoul 03181, Korea; hcfm.shin@samsung.com; 6 Medical Research Institute, Kangbuk Samsung Hospital, Sungkyunkwan University, School of Medicine, 29, Saemunan-ro, Jongno-gu, Seoul 03181, Korea

**Keywords:** cigarette smoking, microbiota, gastrointestinal microbiome, 16S rRNA

## Abstract

There have been few large-scale studies on the relationship between smoking and gut microbiota. We investigated the relationship between smoking status and the composition of gut microbiota. This was a population-based cross-sectional study using Healthcare Screening Center cohort data. A total of 758 men were selected and divided into three groups: never (*n* = 288), former (*n* = 267), and current smokers (*n* = 203). Among the three groups, there was no difference in alpha diversity, however, Jaccard-based beta diversity showed significant difference (*p* = 0.015). Pairwise permutational multivariate analysis of variance (PERMANOVA) tests between never and former smokers did not show a difference; however, there was significant difference between never and current smokers (*p* = 0.017) and between former and current smokers (*p* = 0.011). Weighted UniFrac-based beta diversity also showed significant difference among the three groups (*p* = 0.038), and pairwise PERMANOVA analysis of never and current smokers showed significant difference (*p* = 0.01). In the analysis of bacterial composition, current smokers had an increased proportion of the phylum Bacteroidetes with decreased Firmicutes and Proteobacteria compared with never smokers, whereas there were no differences between former and never smokers. In conclusion, gut microbiota composition of current smokers was significantly different from that of never smokers. Additionally, there was no difference in gut microbiota composition between never and former smokers.

## 1. Introduction

Cigarette smoking has been objectively reported to be associated with lung cancer and has been revealed to be associated with several diseases [1]. Cigarette smoke is a complex mixture of an estimated 7357 chemical compounds and is inhaled into the lung as aerosol particles or free in a gaseous state; it causes more than 480,000 deaths annually in the United States alone [1,2]. Nonetheless, cigarettes are still legally sold in many countries around the world, and a large number of people are exposed to second-hand smoke daily [3]. Cigarette smoking is known to affect not only the tissues and organs of the human body, but also the gut microbiota, which is the community of microorganisms in the gastrointestinal tract [4,5,6,7].

Gut microbiota maintains homeostasis with a mucosal barrier and the immune system in health status and plays an important role in metabolism, modulation of immune response, and protection from pathologic bacteria [7,8,9,10]. In addition, gut microbiota has been reported to be associated with several diseases such as inflammatory bowel diseases, obesity, cardiovascular diseases, cancers, and rheumatoid arthritis [11,12,13]; it is also known to be affected by various factors including host sex, age, diet, antibiotics, other drugs, genetics, and the local environment [14]. Among these factors, cigarette smoking was reported to be associated with *Clostridium difficile* infection and the gut microbiota of inflammatory bowel diseases [15,16]. Biedermann et al. reported changes in gut the microbiota at 4 weeks and 8 weeks after 10 smokers quit smoking [6]. In animal models, it has also been shown that smoking could change the intestinal microbiota though various mechanisms such as changes in mucus and the gut immune system [4,14].

To our knowledge, all previous studies have been carried out with only a small number of subjects or did not fully exclude factors that affect gut microbiota such as antibiotics, diseases, and cholesterol-lowering agents [6,15,17,18]. Therefore, we investigated the relationship between smoking status and gut microbiota in a large number of subjects after eliminating many other factors affecting gut microbiota.

## 2. Materials and Methods

### 2.1. Study Population and Study Design

**Figure 1:**
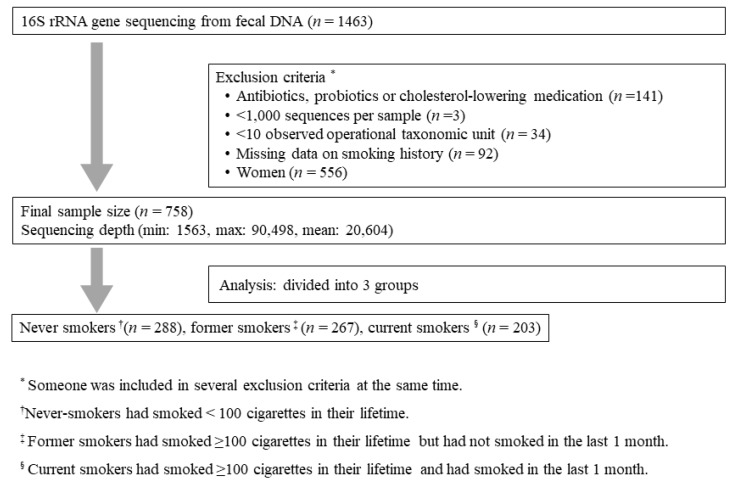
Flow of study.

Participants were recruited from the Kangbuk Samsung Health Study, which is a cohort study of Korean men and women who undergo comprehensive annual or biennial examinations at the Kangbuk Samsung Hospital Healthcare Screening Center in South Korea. Fecal samples were collected from 1463 participants between the ages of 23 and 78 years between June 2014 and September 2014. Among them, 758 men were included in this study (Figure 1). Women were excluded since the percentage of current smokers was too low (12/556). Subjects who had taken medication which could affect gut microbiota such as antibiotics, probiotics and cholesterol-lowering medication, were excluded. Enrolled subjects were divided into three groups: never smokers, former smokers, and current smokers. Demographic characteristics, laboratory results, dietary data, smoking history, and medical history were evaluated. Medical, smoking history, and dietary data were obtained through questionnaires.

### 2.2. DNA Extraction from Fecal Samples and Sequencing of the Bacterial 16S rRNA Gene

Fecal samples were immediately frozen at −20 °C after defecation and were placed at −70 °C within 24 h. Within one month, DNA extraction from fecal samples was performed using the MOBio PowerSoil^®^ DNA Isolation Kit (MO BIO Laboratories, Carlsbad, CA, USA) according to the manufacturer’s instructions. Amplification and sequencing were performed as described previously for analysis of bacterial communities [19]. The genomic DNA was amplified using fusion primers targeting the variable V3 and V4 regions of the *16S rRNA* gene with indexing barcodes. All samples were pooled for sequencing on the Illumina Miseq platform (Illumina, San Diego, CA, USA) according to the manufacturer’s specifications [20,21].

### 2.3. 16S rRNA Gene Compositional Analysis

Quality filtering, chimera removal, and de novo operational taxonomic unit (OTU) clustering were carried out using the UPARSE pipeline [22], which identifies highly accurate OTUs from amplicon sequencing data. The reads were dereplicated, sorted, and clustered into candidate OTUs while removing chimeric OTUs. The taxonomic assignment for OTU was annotated by Ribosomal Database Project reference (version 16) with an identity threshold of 97% using the UTAX command in the UPARSE pipeline. Finally, 29,745,401 reads/208 OTUs with a mean of 20,331 sequences per fecal sample were included for QIIME analysis (http://qiime.org/). Supplementary data include commands used for the UPARSE pipeline and QIIME2.

### 2.4. Statistical Analysis

All continuous values are described as mean ± standard deviation, and categorical values are reported as numbers and percentages. The t test, analysis of variance and the chi-squared test was used for analysis of baseline characteristics among groups. These basic statistical analyses were performed using SPSS version 23 (IBM Inc., Chicago, IL, USA). Exploratory and differential microbial composition analyses were conducted in QIIME2 (version 2017.09) [23]. For the diversity analysis, we rarefied the data to 1000 sequences per sample. Alpha diversity based on identified OTU was estimated using the Shannon index, which means diversity by accounting for evenness and abundance in gut microbial taxa in each sample [24]. The Kruskal–Wallis test and the Mann–Whitney U test were used to estimate the median of difference among groups. Beta diversity was used to analyze the dissimilarity among the groups’ membership and structure. Among beta diversity, Jaccard-based diversity was to identify compositional differences and weighted UniFrac-based diversity to identify phylogenetic abundance differences [10,25]. Permutational multivariate analysis of variance (PERMANOVA) was used to test of significance among groups. The comparison between the two groups within the three groups was measured using pairwise PERMANOVA that were measured in conjunction with the PERMANOVA [23]. Significant difference in microbial taxa abundance between two groups was analyzed using the analysis of composition of microbiome (ANCOM) in the QIIME2 [26]. ANCOM compares the relative abundance of taxa between two groups by log-ratio of abundance of each taxon to the abundance of all the remaining taxa one at a time. We performed ANCOM as the default setting in QIIME2 and the final significance expressed in empirical distribution of W. The resulting *p*-values were corrected for multiple comparisons on each phylogenetic level using the Benjamini–Hochberg correction (FDR). A *p*-value < 0.05 was considered statistically significant.

### 2.6. Ethics Approval and Consent to Participate

Ethical approval for the phenotype, genotype and microbiota studies within the Kangbuk Samsung Cohort Study (KSCS) was provided by the Institutional Review Board of Kangbook Samsung Hospital (KBSMC 2013-01-245-12) and Ewha Mokdong Hospital (EUMC 2017-08-037-001), Seoul, Korea. Written consent was obtained from all participants. Research was carried out in accordance with the Helsinki Declaration.

### 2.7. Availability of Data and Materials

Individual-level 16S sequence data for 1463 samples within this study are available through the CODA (Clinical and Omics data archives, http://coda.nih.go.kr/coda/frt/index.do) repository at the KNIH (Korea National Institute of Health), under accession numbers R000635.

All remaining 16S, genotype and phenotype data in this study are available upon request through application to the KSCS data access committee.

## 3. Results

### 3.1. Baseline Characteristics of the Subjects

The baseline characteristics of enrolled subjects are described in Table 1. Among the total 758 men, 288 (38.0%) were never smokers, 267 (35.2%) were former smokers, and 203 (26.8%) were current smokers. Mean age (*p* < 0.001), prevalence of diabetic mellitus (*p* = 0.014), and smoking variables were significantly different among three groups (*p* < 0.001). In the pulmonary function, there were no differences in forced vital capacity (FVC) or in forced expiratory volume in one second (FEV_1_) among groups. While the mean values of FEV_1_/FVC as an index of obstructive lung disease were normal in all groups, there was a difference among groups (*p* < 0.001). There was no difference in the mean daily amount of cigarette smoking between former smokers and current smokers (14.5 cigarette/day vs. 14.3 cigarette/day, *p* = 0.792). Although creatinine level and estimated glomerular filtration rate (eGFR) showed differences among groups, the prevalence of kidney disease was not different. Mean body mass index (BMI), muscle mass, and fat mass did not differ significantly among groups. Other variables including nutritional composition based on questionnaires of subjects did not show significant differences among groups.

### 3.2. Diversity within and among Groups

The richness and evenness of gut microbial taxa within never smokers, former smokers, and current smokers, as measured using the Shannon index of alpha diversity, did not show significant differences (never smokers vs. former smoker vs. current smokers, *p* = 0.179) (Supplement Figure S1).

**Figure 2:**
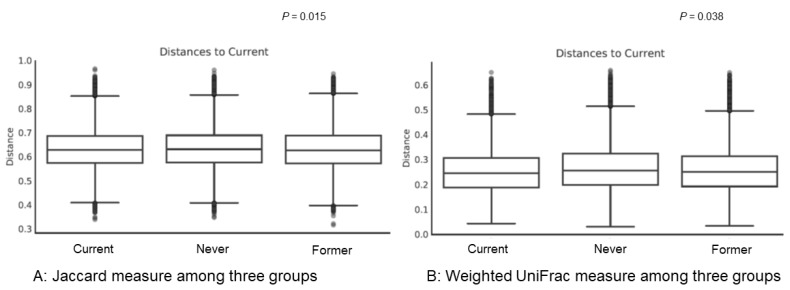
Beta diversity among groups. (**A**) Results of beta diversity using Jaccard measure among current smokers, former smokers, and never smokers; (**B**) results of beta diversity using weighted UniFrac measure among current smokers, former smokers, and never smokers. The *y*-axes represent the distance of each group to the current group (baseline). The line in each box means the median of data. In both Jaccard measure and Weight UniFrac measure, *p*-values among three groups were estimated using permutational multivariate analysis of variance (PERMANOVA). PERMANOVA of the diversity analysis was calculated with the 999 Monte Carlo permutation and Benjamini–Hochberg correction (FDR).

However, there were significant differences in beta diversity indices, which suggests diversity among the three groups. Results of Jaccard-based diversity showed significant compositional differences among the three groups (Figure 2A, *p* = 0.015). In pairwise PERMANOVA, there was no difference between never smokers and former smokers (*p* = 0.523); however, there was a significant difference between never smokers and current smokers (*p* = 0.017) and between former smokers and current smokers (*p* = 0.011). Results of weighted UniFrac-based diversity showed significant phylogenetic abundance differences among the three groups (Figure 2B, *p* = 0.038). In pairwise PERMANOVA, analysis of never smokers and current smokers showed significant differences (*p* = 0.001); however, analysis of never and former smokers and analysis of former and current smokers showed no significant differences (*p* = 0.258 and *p* = 0.115, respectively).

### 3.3. Analysis of Microbiota Composition

**Figure 3:**
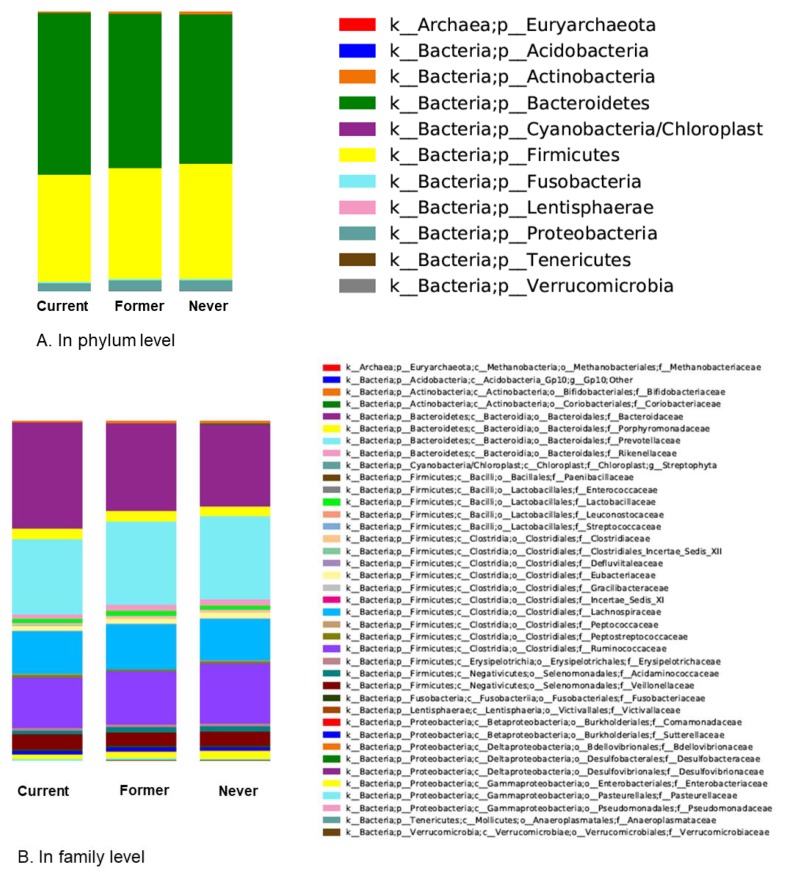
Bar chart of proportional abundance of phylum (**A**) and family (**B**) levels in the three smoking categories.

**Figure 4:**
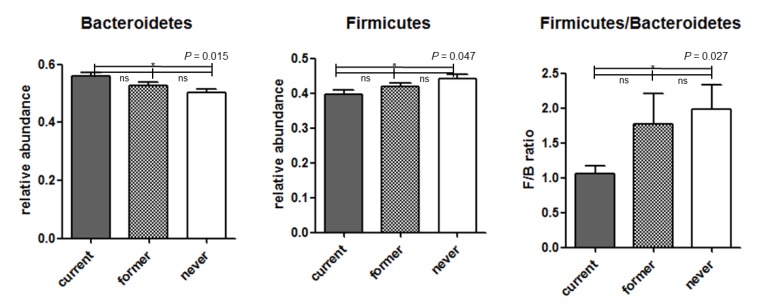
Comparison among groups of phylum Bacteroidetes and phylum Firmicutes with high gut microbiota composition. ns, non-significant; *, true values in analysis of composition of microbiomes.

Figure 3 shows taxonomic assignment with compositional changes among the three groups. The proportion differences of phylum Bacteroidetes and phylum Firmicutes, which account for a large proportion of gut microbiota, are shown in Figure 4. Compared with never smokers, current smokers showed a higher relative abundance of phylum Bacteroidetes, a lower relative abundance of phylum Firmicutes, and a lower Firmucutes/Bacteroidetes ratio.

We investigated statistical differences in microbial taxa abundance between two groups using the ANCOM method (Table 2, Supplement Table S1). This analysis of relative abundances on the phylum through genus levels revealed that notably there were far more significant results in the comparison between never and current smokers. Current smokers had a significantly increased proportion of phylum Bacteroidetes and decreased proportions of phylum Firmicutes and phylum Proteobacteria compared to never smokers. There were also many taxonomic differences at the family level (Table 2) as well as the genus level (Supplement Table S1) between never and current smokers. However, former and current smokers showed taxa abundance differences only at the phylum level, but no differences at the family level. Phylum Bacteroidetes and phylum Tenericutes were increased and phylum Verrucomicrobia was decreased in current smokers compared with former smokers. Never and former smokers did not have any difference in ANCOM.

### 3.4. Additional Analysis of Subjects

We divided the subjects into two: (current smokers and current non-smokers (both never and former smokers), and performed additional analyses (Supplement Table S2, Supplement Figures S2–S5) since our analysis between former and never smokers did not show significant differences, and a previous large study of smoking and oral microbiota reported differences between current and current non-smokers [27]. There was no difference in alpha diversity (Supplement Figure S2, current non-smokers vs. current smokers, *p* = 0.066). However, beta diversity measures showed significant differences in both Jaccard and weighted UniFrac measure methods (Supplement Figure S3, *p* = 0.006 and *p* = 0.021, respectively). In the ANCOM method, current smokers had a significantly increased proportion of phylum Bacteroidetes compared to current non-smokers (Supplement Table S1).

## 4. Discussion

In this large scale-study, we found that gut microbiota was associated with smoking status in men. Particularly, at the phylum level, current smokers had a higher proportion of Bacteroidetes in their gut microbiota compared with never and former smokers. In addition, current smokers had lower proportions of Firmicutes and Proteobacteria compared with never smokers. However, the composition of gut microbiota between never and former smokers did not show significant differences, which suggests that, if smokers quit smoking, gut microbiota composition is likely to recover to pre-smoking status.

There have been few studies on the relationship between smoking and human gut microbiota, and most of these studies have been performed in specific patient groups such as in individuals with Crohn’s disease [28,29]. A study by Biedermann et al. seems to be the only one that examined the association between smoking and gut microbiota in smokers without specific diseases [6]. However, they described the longitudinal changes of gut microbiota at the 4th and 8th weeks after smoking cessation only in 10 smokers. In that study, smoking cessation induced an increase in Firmicutes and Actinobacteria and a decrease in Bacteroidetes and Proteobacteria [6]. For Firmicutes and Bacteriodetes, our results seem to be comparable to those of Biedermann’s study [6]. However, which phylum is affected by smoking requires further research, because unlike our study, that of Biedermann et al. compared gut microbiota before and after quitting smoking only in 10 subjects, and most of them had increased BMI after smoking cessation [6]. It is known that persons with higher BMI have increased Firmicutes and decreased Bacteroidetes in the gut compared to those with normal BMI [13,30,31]. Therefore, changes in gut microbiota of individuals in Biedermann’s study [6] might be associated with weight gain after smoking cessation as well as with smoking itself. In contrast to Biedermann’s longitudinal study [6], our study was a cross-sectional study comparing the gut microbiota of current smokers, former smokers, and never smokers, and our three groups did not differ in terms of BMI or nutrient intake. In our study, never smokers showed an increase in Firmicutes and Proteobacteria and a decrease in Bacteroidetes compared to current smokers. Similarly, other cross-sectional studies on smoking and gut microbiota in patients with Crohn’s disease also showed an increase in Bacteroidetes in the gut microbiota of smokers [28,29]. Therefore, in most studies, including our study, it was shown that the proportion of gut Bacteroidetes tends to increase with current smoking.

In our study, gut microbiota composition of former smokers was different from that of current smokers at the phylum level, but it was not different from that of never smokers. Former smokers in our study likely correspond to the smoking cessation group in the Bierderman study. According to Bierderman, intestinal bacterial composition and diversity began to change from the fourth week after smoking cessation [6]. In the current study, we defined former smokers as those who had not smoked during the last one month. However, the average smoking cessation period of former smokers is estimated to be approximately 6 years on average. This is because, when they were smoking, the average daily smoking amount of former smokers was almost the same as that of current smokers (14.5 cigarette/day vs. 14.3 cigarette/day, *p* = 0.792), but the average smoking period of former smokers was about 6 years shorter than that of current smokers. In other words, the increased alpha diversity at the fourth week after smoking cessation, as observed in Bierderman’s study, might have disappeared in former smokers of our study, who had a longer period of smoking cessation. In addition, as the smoking cessation period gets longer, the composition of gut microbiota of former smokers is likely to be more similar to that of never smokers than that of current smokers. However, further study is needed on the timing of changes in gut microbiota composition after smoking cessation. Additionally, similar to our study, a comparative large-scale cross-sectional study on the relationship between smoking and oral bacterial composition showed that the oral bacterial composition of current smokers was different from that of never smokers and former smokers, but there was no difference between former smokers and never smokers [27]. In fact, the effects of smoking cessation on health can be expected to be as good as the reduction of cardiovascular disease, even over a relatively short period of smoking cessation except for lung cancer [32].

Although the biological mechanism between smoking and gut environment is not yet exactly known, several researchers have reported animal studies on the effects of smoking on gut microbiota [4,33,34]. Smoking seems to affect bowel mucosa and mucin expression [4,35]. For example, nicotine, among the complex mixtures in cigarettes, decreased prostaglandin E2 production, increased nitric oxide synthase activity, and finally led to progression of jejunitis in murine models [36,37]. Based on nicotine effects on the central nervous system and the effects of gut microbiota on neural activity, one study showed that nicotine may alter the microbiota by signaling the gut–brain axis in a murine model [38]. Gut microbiota is also known to play a role in metabolism of nutrients, drugs, and potentially toxic compounds, and in the maintenance of structural integrity [9,39]. The absorption of several toxic chemical compounds in cigarette smoking could change metabolism and alter the composition of gut microbiota. Since microbiota have recently been revealed to be associated with host immune function [8], cigarette smoking, which can affect immune function, may indirectly affect the composition of gut microbiota [40]. Actually, short chain fatty acids (SCFA), which are associated with regulation of immune cell function, are known to be associated with members of the phylum Bacteroidetes [41]. Furthermore, Crohn’s disease, a type of inflammatory bowel disease, is known to be affected by cigarette smoking, though it is unclear which factors of smoking contribute to the pathogenesis of Crohn’s disease. Smokers with Crohn’s disease showed changes in the composition of gut microbiota, especially increased levels of Bacteroidetes [18,28]. Other studies have reported that gut microbiota is associated with human metabolic diseases [42,43], and the Firmicutes/Bacteroidetes ratio is associated with predisposition to disease states and obesity [30,31,44].

The greatest strength of this study is that it clearly demonstrated the relationship between smoking status and gut microbiota. This conclusion is unlikely to be controversial, as our study is not only the largest study, but also excluded the effects of medication affecting gut microbiota such as probiotics, antibiotics, and cholesterol-lowering medication. In addition, the long-term effects of smoking cessation on gut microbiota can be supposed through a large number of former smokers. Nevertheless, our study has several limitations. First, it was a cross-sectional study, which cannot determine causality. Second, we have a technical limitation from 16S amplicon-based sequencing data which can introduce biases through polymerase chain reaction (PCR) amplification steps, and resolve only genus level as a maximum. Third, although we adjusted some medications, there are the effects of potential confounders such as diet and other medications which we have not noticed. Fourth, we studied only men since most women in this cohort were never smokers; the rates of men and women smoking in Korea are 42.1% and 6.2%, respectively [45]. Finally, although our study showed many taxonomic differences at the family level between never and current smokers, this study was not enough to discuss the family level since studies about human and gut microbiota are few. With reference to a recently reported study about smoking and gut microbiota, most family levels of gut microbiota related with smoking were revealed in animal experiments [46]. Therefore, further studies into smoking and human gut microbiota are needed.

## 5. Conclusions

The composition of gut microbiota is associated with smoking status in men; in particular, the gut microbiota of current smokers was composed of significantly higher levels of Bacteroidetes. Also, if smokers quit smoking for a long period of time, their gut microbiota may return to the microbiota of never smokers. Therefore, for diseases related to the alteration of gut microbiota, smoking cessation is likely to be one of the best treatments.

## Figures and Tables

**Table 1:** Baseline characteristics of the study population.

Variable	Never (*N* = 288)	Former (*N* = 267)	Current (*N* = 203)	*p*-Value
Age, year	44.2 ± 9.1	47.2 ± 8.5	45.7 ± 8.2	<0.001
BMI, kg/m^2^	24.7 ± 3.0	24.6 ± 2.6	24.8 ± 3.1	0.823
Muscle mass, kg	52.8 ± 5.8	52.5 ± 5.4	53.2 ± 6.0	0.455
Fat mass, kg	17.3 ± 5.7	17.1 ± 4.9	17.2 ± 5.7	0.864
Smoking history				
Smoking amount, pack-years	0	12.9 ± 10.2	17.6 ± 12.4	<0.001
Smoking amount, cigarette/day	0	14.5 ± 7.0	14.3 ± 7.2	<0.001
Smoking duration, years	0	16.2 ± 9.0	22.9 ± 8.7	<0.001
Creatinine, mg/dL	0.99 ± 0.12	1.0 ± 0.11	0.97 ± 0.13	0.011
eGFR, MDRD, /mL min/1.73 m^2^	88.8 ± 13.1	86.9 ± 11.5	91.5 ± 14.6	0.001
Iron, μg/dL	127.0 ± 37.9	127.0 ± 39.3	127.9 ± 38.7	0.972
Ferritin, ng/mL	203 ± 111.8	218.5 ± 134.1	224.8 ± 164.4	0.176
C-reactive protein, mg/dL	0.11 ± 0.17	0.12 ± 0.21	0.12 ± 0.19	0.754
Comorbidities				
Diabetes mellitus	11 (3.8)	27 (10.1)	15 (7.4)	0.014
Hypertension	53 (18.4)	60 (22.5)	36 (17.7)	0.500
COPD	8 (2.8)	14 (5.2)	15 (7.4)	0.065
Liver disease *	46 (16)	58 (21.7)	33 (16.3)	0.160
Dyslipidemia	54 (18.8)	55 (20.6)	41 (20.2)	0.858
Kidney disease ^†^	10 (3.5)	14 (5.2)	7 (3.4)	0.500
Spirometry				
FVC, % predicted	89.2 ± 9.4	89.6 ± 8.8	90.2 ± 9.5	0.489
FEV_1_, % predicted	89.6 ± 9.3	89.0 ± 9.9	88.7 ± 10.7	0.583
FEV_1_/FVC (%)	81.4 ± 5.9	79.5 ± 5.9	79.3 ± 6.9	<0.001
Nutrition				
Total energy, kcal/day	1485.6 ± 667.5	1417.6 ± 570.4	1523.4 ± 634.9	0.266
Total protein, g/day	51.2 ± 28.4	48.6 ± 22.1	53.1 ± 25.4	0.251
Total fat, g/day	31.0 ± 22.2	26.8 ± 15.8	30.8 ± 19.3	0.054
Total carbohydrate, g/day	246.1 ± 105.0	241.4 ± 101.9	253.7 ± 111.3	0.557
Total calcium, mg/day	314.2 ± 215.7	287.5 ± 167.9	306.6 ± 186.5	0.346
Total phosphorus, mg/day	739.8 ± 380.8	700.6 ± 294.4	759.1 ± 344.2	0.252
Total vitamin A, ug/day	309.3 ± 210.6	311.9 ± 218.0	344.8 ± 230.4	0.255
Total sodium, mg/day	1613.4 ± 1032.5	1578.2 ± 987.6	1809.8 ± 1093.8	0.089
Vitamin B1, mg/day	0.87 ± 0.48	0.82 ± 0.41	0.91 ± 0.46	0.176
Vitamin C, mg/day	60.5 ± 46.1	70.0 ± 59.5	66.7 ± 47.2	0.158
Folate, mg/day	141.8 ± 89.4	145.7 ± 96.6	152.8 ± 93.5	0.53
Retinol, ug/day	75.4 ± 63.8	67.0 ± 51.9	72.9 ± 60.6	0.327
Fiber, g/day	3.5 ± 2.0	3.7 ± 2.1	3.8 ± 2.1	0.42
Cholesterol, mg/day	171.8 ± 153.1	171.3 ± 138.0	182.6 ± 151.5	0.734

Values represented as mean ± standard deviation or *N* (%); BMI, body mass index; COPD, chronic obstructive pulmonary disease; FVC, forced vital capacity; FEV_1_, forced expiratory volume in one second; eGFR, estimated glomerular filtration rate; MDRD, modification of diet in renal disease * liver disease including hepatitis B, hepatitis C, liver cirrhosis, and fatty liver; ^†^ kidney disease including chronic kidney disease, ureter stones, and benign prostate hypertrophy; *p*-value for difference between groups by analysis of variance test (ANOVA) for continuous variables and the chi-squared test for categorical variables.

**Table 2:** Results of analysis of microbiome composition.

Level	Taxonomic Assignment	w
**Never vs. current**		
**Phylum**	k__Bacteria;p__Bacteroidetes (increased in current smokers) *	2
	k__Bacteria;p__Firmicutes (decreased in current smokers)	1
	k__Bacteria;p__Proteobacteria (decreased in current smokers)	2
**Family**	k__Bacteria;p__Bacteroidetes;c__Bacteroidia;o__Bacteroidales;f__Bacteroidaceae * (increased in current smokers) *	5
	k__Bacteria;p__Bacteroidetes;c__Bacteroidia;o__Bacteroidales;f__Porphyromonadaceae * (increased in current smokers) *	1
	k__Bacteria;p__Firmicutes;c__Bacilli;o__Lactobacillales;f__Lactobacillaceae * (increased in current smokers) *	3
	k__Bacteria;p__Firmicutes;c__Clostridia;o__Clostridiales;f__Clostridiales_Incertae_Sedis_XII (decreased in current smokers)	3
	k__Bacteria;p__Firmicutes;c__Clostridia;o__Clostridiales;f__Gracilibacteraceae (decreased in current smoker)s	1
	k__Bacteria;p__Firmicutes;c__Clostridia;o__Clostridiales;f__Peptococcaceae (decreased in current smokers)	6
	k__Bacteria;p__Firmicutes;c__Clostridia;o__Clostridiales;f__Ruminococcaceae (decreased in current smokers)	1
	k__Bacteria;p__Fusobacteria;c__Fusobacteriia;o__Fusobacteriales;f__Fusobacteriaceae (decreased in current smokers)	3
	k__Bacteria;p__Proteobacteria;c__Betaproteobacteria;o__Burkholderiales;f__Comamonadaceae (decreased in current smokers)	1
	k__Bacteria;p__Proteobacteria;c__Gammaproteobacteria;o__Enterobacteriales;f__Enterobacteriaceae (decreased in current smokers)	2
**Former vs. current**		
**Phylum**	k__Bacteria;p__Bacteroidetes (increased in current smokers) *	1
	k__Bacteria;p__Tenericutes (increased in current smokers) *	1
	k__Bacteria;p__Verrucomicrobia (decreased in current smokers)	2
**Family**	None	
**Never vs. former**		
**Phylum**	None	
**Family**	None	

k, kingdom; p, phylum; c, class; o, order; f, family; * marker bacteria are increased in current smokers.

## Supplementary Materials

The following are available online at http://www.mdpi.com/2077-0383/7/9/282/s1, Figure S1. Results of alpha diversity using the Shannon index among current smokers, former smokers, and never smokers; Figure S2. Results of alpha diversity using the Shannon index between current smokers and current non-smokers; Figure S3. Results of beta diversity between current smokers and current non-smokers; Figure S4. Bar chart of proportional abundance of phylum (A) and family (B) levels between current smokers and current non-smokers; Figure S5. Comparison among groups of phylum Bacteroidetes and phylum Firmicutes with a high proportion of gut microbiota composition between current smokers and current non-smokers; Table S1. Results of analysis of composition microbiomes; Table S2. Baseline characteristic of study population between Current non-smoker and current smokers.

## References

[1] Alberg A.J., Shopland D.R., Cummings K.M. (2014). The 2014 surgeon general’s report: Commemorating the 50th anniversary of the 1964 report of the advisory committee to the us surgeon general and updating the evidence on the health consequences of cigarette smoking. Am. J. Epidemiol..

[2] Centers for Disease Control and Prevention (US), National Center for Chronic Disease Prevention and Health Promotion (US), Office on Smoking and Health (US) (2010). Publications and Reports of the Surgeon General. How Tobacco Smoke Causes Disease: The Biology and Behavioral Basis for Smoking-Attributable Disease: A Report of the Surgeon General.

[3] Bilano V., Gilmour S., Moffiet T., d’Espaignet E.T., Stevens G.A., Commar A., Tuyl F., Hudson I., Shibuya K. (2015). Global trends and projections for tobacco use, 1990–2025: An analysis of smoking indicators from the who comprehensive information systems for tobacco control. Lancet.

[4] Allais L., Kerckhof F.M., Verschuere S., Bracke K.R., De Smet R., Laukens D., Van den Abbeele P., De Vos M., Boon N., Brusselle G.G. (2016). Chronic cigarette smoke exposure induces microbial and inflammatory shifts and mucin changes in the murine gut. Environ. Microbiol..

[5] Biedermann L., Brulisauer K., Zeitz J., Frei P., Scharl M., Vavricka S.R., Fried M., Loessner M.J., Rogler G., Schuppler M. (2014). Smoking cessation alters intestinal microbiota: Insights from quantitative investigations on human fecal samples using fish. Inflamm. Bowel Dis..

[6] Biedermann L., Zeitz J., Mwinyi J., Sutter-Minder E., Rehman A., Ott S.J., Steurer-Stey C., Frei A., Frei P., Scharl M. (2013). Smoking cessation induces profound changes in the composition of the intestinal microbiota in humans. PLoS ONE.

[7] Browne H.P., Neville B.A., Forster S.C., Lawley T.D. (2017). Transmission of the gut microbiota: Spreading of health. Nat. Rev. Microbiol..

[8] Thomas S., Izard J., Walsh E., Batich K., Chongsathidkiet P., Clarke G., Sela D.A., Muller A.J., Mullin J.M., Albert K. (2017). The host microbiome regulates and maintains human health: A primer and perspective for non-microbiologists. Cancer Res..

[9] Claus S.P., Guillou H., Ellero-Simatos S. (2016). The gut microbiota: A major player in the toxicity of environmental pollutants?. NPJ Biofilms Microbiomes.

[10] Faith J.J., Guruge J.L., Charbonneau M., Subramanian S., Seedorf H., Goodman A.L., Clemente J.C., Knight R., Heath A.C., Leibel R.L. (2013). The long-term stability of the human gut microbiota. Science.

[11] Zhang H., Diao H., Jia L., Yuan Y., Thamm D.H., Wang H., Jin Y., Pei S., Zhou B., Yu F. (2017). Proteus mirabilis inhibits cancer growth and pulmonary metastasis in a mouse breast cancer model. PLoS ONE.

[12] Gilbert J.A., Quinn R.A., Debelius J., Xu Z.Z., Morton J., Garg N., Jansson J.K., Dorrestein P.C., Knight R. (2016). Microbiome-wide association studies link dynamic microbial consortia to disease. Nature.

[13] Ley R.E. (2010). Obesity and the human microbiome. Curr. Opin. Gastroenterol..

[14] Capurso G., Lahner E. (2017). The interaction between smoking, alcohol and the gut microbiome. Best Pract. Res. Clin. Gastroenterol..

[15] Rogers M.A., Greene M.T., Saint S., Chenoweth C.E., Malani P.N., Trivedi I., Aronoff D.M. (2012). Higher rates of clostridium difficile infection among smokers. PLoS ONE.

[16] Frank D.N., St Amand A.L., Feldman R.A., Boedeker E.C., Harpaz N., Pace N.R. (2007). Molecular-phylogenetic characterization of microbial community imbalances in human inflammatory bowel diseases. Proc. Natl. Acad. Sci. USA.

[17] Quigley E.M.M. (2017). Gut microbiome as a clinical tool in gastrointestinal disease management: Are we there yet?. Nat. Rev. Gastroenterol. Hepatol..

[18] Parkes G.C., Whelan K., Lindsay J.O. (2014). Smoking in inflammatory bowel disease: Impact on disease course and insights into the aetiology of its effect. J. Crohns Colitis.

[19] Kim H.N., Yun Y., Ryu S., Chang Y., Kwon M.J., Cho J., Shin H., Kim H.L. (2018). Correlation between gut microbiota and personality in adults: A cross-sectional study. Brain Behav. Immun..

[20] Fadrosh D.W., Ma B., Gajer P., Sengamalay N., Ott S., Brotman R.M., Ravel J. (2014). An improved dual-indexing approach for multiplexed 16s rrna gene sequencing on the illumina miseq platform. Microbiome.

[21] Kozich J.J., Westcott S.L., Baxter N.T., Highlander S.K., Schloss P.D. (2013). Development of a dual-index sequencing strategy and curation pipeline for analyzing amplicon sequence data on the miseq illumina sequencing platform. Appl. Environ. Microbiol..

[22] Edgar R.C. (2013). Uparse: Highly accurate otu sequences from microbial amplicon reads. Nat. Methods.

[23] Navas-Molina J.A., Peralta-Sanchez J.M., Gonzalez A., McMurdie P.J., Vazquez-Baeza Y., Xu Z., Ursell L.K., Lauber C., Zhou H., Song S.J. (2013). Advancing our understanding of the human microbiome using QIIME. Methods Enzymol..

[24] Chao A., Shen T.-J. (2003). Nonparametric estimation of shannon’s index of diversity when there are unseen species in sample. Environ. Ecol. Stat..

[25] Lozupone C., Lladser M.E., Knights D., Stombaugh J., Knight R. (2011). Unifrac: An effective distance metric for microbial community comparison. ISME J..

[26] Mandal S., Van Treuren W., White R.A., Eggesbo M., Knight R., Peddada S.D. (2015). Analysis of composition of microbiomes: A novel method for studying microbial composition. Microb. Ecol. Health Dis..

[27] Wu J., Peters B.A., Dominianni C., Zhang Y., Pei Z., Yang L., Ma Y., Purdue M.P., Jacobs E.J., Gapstur S.M. (2016). Cigarette smoking and the oral microbiome in a large study of american adults. ISME J..

[28] Opstelten J.L., Plassais J., van Mil S.W.C., Achouri E., Pichaud M., Siersema P.D., Oldenburg B., Cervino A.C.L. (2016). Gut microbial diversity is reduced in smokers with Crohn’s disease. Inflamm. Bowel Dis..

[29] Benjamin J.L., Hedin C.R., Koutsoumpas A., Ng S.C., McCarthy N.E., Prescott N.J., Pessoa-Lopes P., Mathew C.G., Sanderson J., Hart A.L. (2012). Smokers with active Crohn’s disease have a clinically relevant dysbiosis of the gastrointestinal microbiota. Inflamm. Bowel Dis..

[30] Ley R.E., Turnbaugh P.J., Klein S., Gordon J.I. (2006). Microbial ecology: Human gut microbes associated with obesity. Nature.

[31] Koliada A., Syzenko G., Moseiko V., Budovska L., Puchkov K., Perederiy V., Gavalko Y., Dorofeyev A., Romanenko M., Tkach S. (2017). Association between body mass index and firmicutes/bacteroidetes ratio in an adult ukrainian population. BMC Microbiol..

[32] Jha P., Peto R. (2014). Global effects of smoking, of quitting, and of taxing tobacco. N. Engl. J. Med..

[33] Tomoda K., Kubo K., Asahara T., Andoh A., Nomoto K., Nishii Y., Yamamoto Y., Yoshikawa M., Kimura H. (2011). Cigarette smoke decreases organic acids levels and population of bifidobacterium in the caecum of rats. J. Toxicol. Sci..

[34] Wang H., Zhao J.X., Hu N., Ren J., Du M., Zhu M.J. (2012). Side-stream smoking reduces intestinal inflammation and increases expression of tight junction proteins. World J. Gastroenterol..

[35] Zijlstra F.J., Srivastava E.D., Rhodes M., van Dijk A.P., Fogg F., Samson H.J., Copeman M., Russell M.A., Feyerabend C., Williams G.T. (1994). Effect of nicotine on rectal mucus and mucosal eicosanoids. Gut.

[36] Verschuere S., Bracke K.R., Demoor T., Plantinga M., Verbrugghe P., Ferdinande L., Lambrecht B.N., Brusselle G.G., Cuvelier C.A. (2011). Cigarette smoking alters epithelial apoptosis and immune composition in murine galt. Lab. Investig..

[37] Eliakim R., Fan Q.X., Babyatsky M.W. (2002). Chronic nicotine administration differentially alters jejunal and colonic inflammation in interleukin-10 deficient mice. Eur. J. Gastroenterol. Hepatol..

[38] Chi L., Mahbub R., Gao B., Bian X., Tu P., Ru H., Lu K. (2017). Nicotine alters the gut microbiome and metabolites of gut-brain interactions in a sex-specific manner. Chem. Res. Toxicol..

[39] Jandhyala S.M., Talukdar R., Subramanyam C., Vuyyuru H., Sasikala M., Reddy D.N. (2015). Role of the normal gut microbiota. World J. Gastroenterol..

[40] Sopori M. (2002). Effects of cigarette smoke on the immune system. Nat. Rev. Immunol..

[41] Corrêa-Oliveira R., Fachi J.L., Vieira A., Sato F.T., Vinolo M.A.R. (2016). Regulation of immune cell function by short-chain fatty acids. Clin. Transl. Immunol..

[42] Johnson E.L., Heaver S.L., Walters W.A., Ley R.E. (2017). Microbiome and metabolic disease: Revisiting the bacterial phylum bacteroidetes. J. Mol. Med..

[43] Morrison D.J., Preston T. (2016). Formation of short chain fatty acids by the gut microbiota and their impact on human metabolism. Gut Microbes.

[44] Mariat D., Firmesse O., Levenez F., Guimaraes V., Sokol H., Dore J., Corthier G., Furet J.P. (2009). The firmicutes/bacteroidetes ratio of the human microbiota changes with age. BMC Microbiol..

[45] Choi S., Kim Y., Park S., Lee J., Oh K. (2014). Trends in cigarette smoking among adolescents and adults in South Korea. Epidemiol. Health.

[46] Savin Z., Kivity S., Yonath H., Yehuda S. (2018). Smoking and the intestinal microbiome. Arch. Microbiol..

