# Supplementary material for: Association between Cigarette Smoking Status and Composition of Gut Microbiota: Population-Based Cross-Sectional Study
Source: J Clin Med. 2018 Sep 14;7(9):282. doi: 10.3390/jcm7090282 (PMC6162563; doi:10.3390/jcm7090282)
Supplement: Supplementary file 1 [file jcm-07-00282-s001.pdf]

Supplement data.

Association between cigarette smoking status and composition of gut microbiota: population based cross-sectional study

Command

# UPARSE

Sequence merge: usearch91 -fastq_mergepairs *R1*.fastq -relabel @ -fastq_maxdiffs 20 -fastq_maxdiffpct 20 -fastq_minmergelen 300 -fastqout merged.fq -threads 10

Filter: usearch91 -fastq_filter merged.fq -fastq_maxee 1.0 -fastaout filtered.fa

Unique sequences: usearch91 -fastx_uniques filtered.fa -fastaout uniques.fa -relabel Uniq -sizeout

OTUs picking: usearch91 -cluster_otus uniques.fa -minsize 2 -otus otus.fa -relabel OTU

Phylogenetic tree: usearch91 -cluster_agg otus.fa -treeout tree.phy

Taxonomy annotation: usearch91 -utax otus.fa -db 16s.udb -strand both -fastaout otus_tax.fa

OTU table: usearch91 -usearch_global merged.fq -db otus_tax.fa -strand plus -id 0.97 -otutabout otutable_tax.txt -biomout otutable_tax.jason

# Diversity

```
qiime diversity core-metrics-phylogenetic \
   --i-phylogeny rooted-tree.qza \
   --i-table table.qza \
   --p-sampling-depth 1000 \
   --m-metadata-file sample-metadata.txt \
   --output-dir core-metrics-results
```

Alpha diversity

```
qiime diversity alpha-group-significance \
   --i-alpha-diversity core-metrics-results/shannon_vector.qza\
   --m-metadata-file sample-metadata.txt \
   --o-visualization core-metrics-results/shannon-group-significance.qzv
```

Beta diversity

```
qiime diversity beta-group-significance \
   --i-distance-matrix core-metrics-results/weighted_unifrac_distance_matrix.qza \
   --m-metadata-file sample-metadata.txt \
```

```
    --m-metadata-category smoking \
    --o-visualization core-metrics-results/weighted-unifrac-significance.qzv \
    --p-pairwise
```

ANCOM

```
qiime feature-table filter-samples \
    --i-table table.qza \
    --m-metadata-file samples-to-keep.txt \
    --o-filtered-table index-filtered-table.qza

qiime composition add-pseudocount \
    --i-table table_gut.qza \
    --o-composition-table comp-gut-table.qza

qiime composition ancom \
    --i-table comp-gut-table.qza \
    --m-metadata-file sample-metadata.txt \
    --m-metadata-category Subject \
    --o-visualization ancom-Subject.qzv

qiime taxa collapse \
    --i-table table_gut.qza \
    --i-taxonomy taxonomy.qza \
    --p-level 2 \
    --o-collapsed-table table_gut_l2.qza

qiime composition add-pseudocount \
    --i-table table_gut_l2.qza \
    --o-composition-table comp-gut-table-l2.qza

qiime composition ancom \
    --i-table comp-gut-table-l2.qza \
    --m-metadata-file sample-metadata.txt \
    --m-metadata-category Subject \
    --o-visualization l2-ancom-Subject.qzv
```

Figure S1 Results of alpha diversity using the Shannon index among current smokers, former smokers, and never smokers. The line in each box means the median of data. *P*-value among three groups were estimated using the Kruskal-Wallis test.

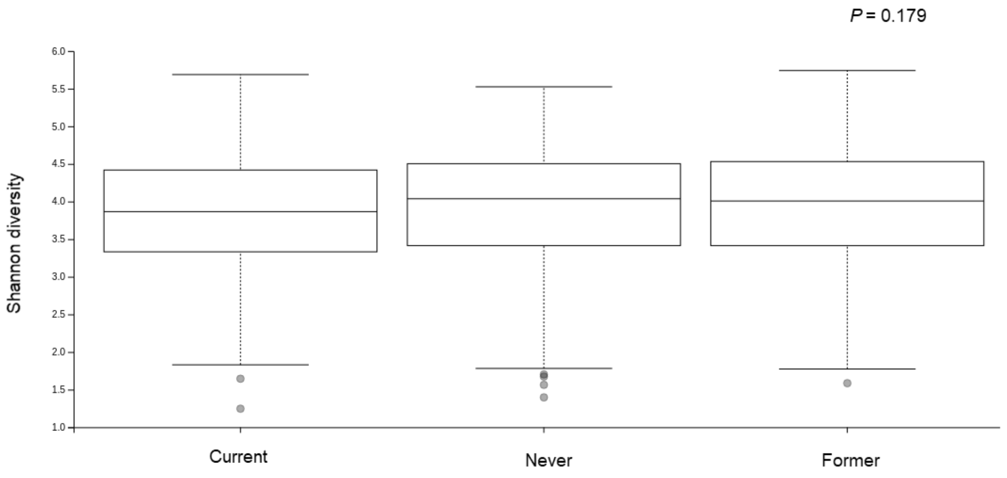

Figure S2 Results of alpha diversity using the Shannon index between current smokers and current non-smokers. The line in each box means the median of data. *P*-value between two groups was estimated using the Mann-Whitney U test.

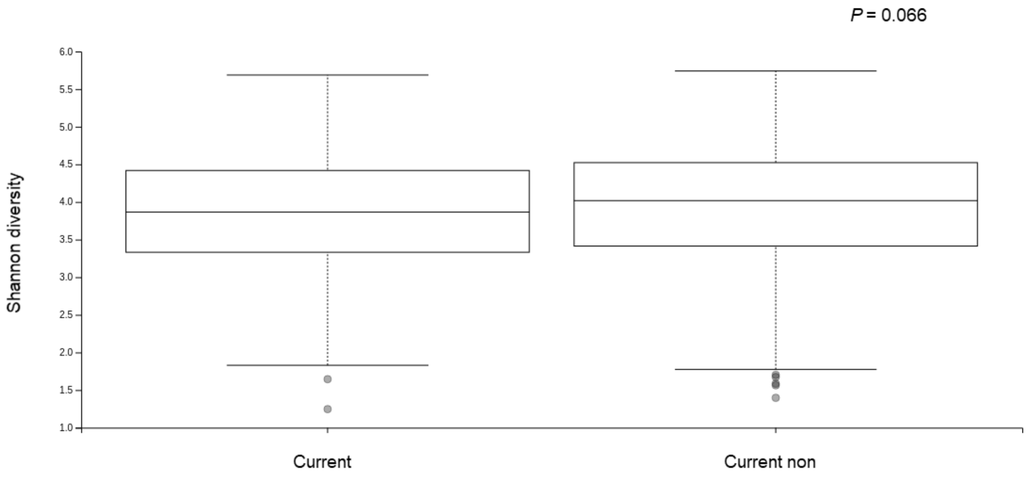

Figure S3 Results of beta diversity between current smokers and current non-smokers

(A) Results of beta diversity using the Jaccard measure between current smokers and current non-smokers. (B) Results of beta diversity using weighted UniFrac measure between current smokers and current non-smokers. The y-axis represents the distance of each group to the current group (baseline). In both Jaccard measure and Weight UniFrac measure, the line in each box means the median of data. In both *P*-values between two groups were estimated using the PERMANOVA. PERMANOVA for the pairwise comparison of the diversity analysis was calculated with 999 Monte Carlo permutation and Benjamini-Hochberg correction (FDR).

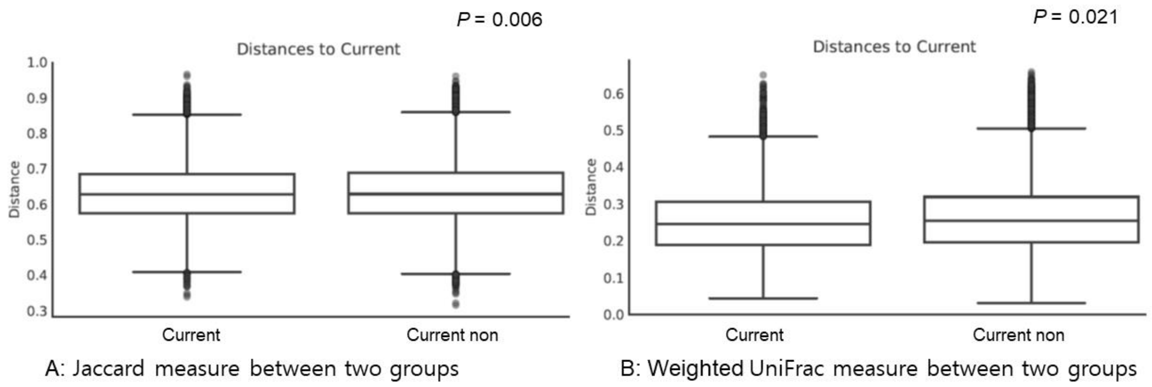

Figure S4 Bar chart of proportional abundance of phylum (A) and family (B) levels between current smokers and current non-smokers

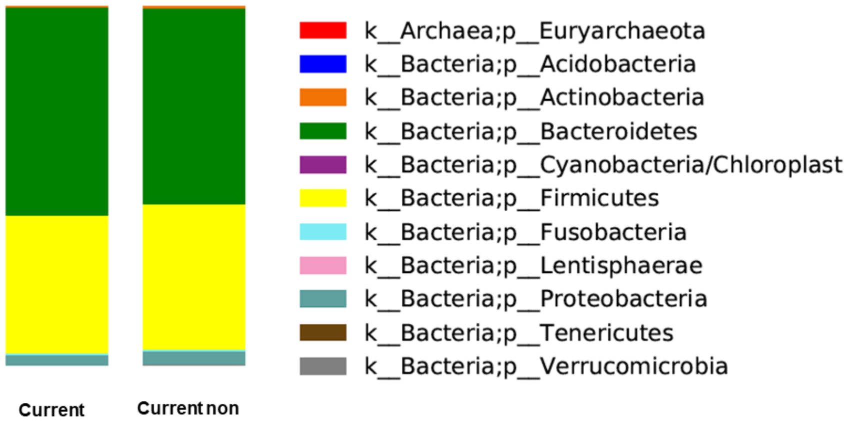

A. In phylum level

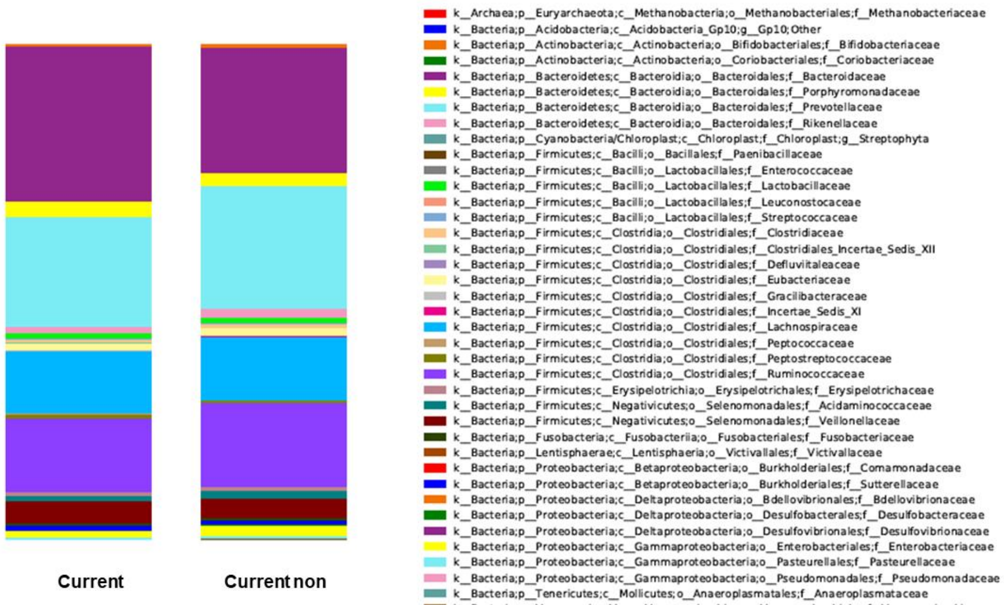

B. In family level

Figure S5 Comparison among groups of phylum Bacteroidetes and phylum Firmicutes with a high proportion of gut microbiota composition between current smokers and current non-smokers. ns, non-significant; *or**, true values in analysis of composition of microbiomes.

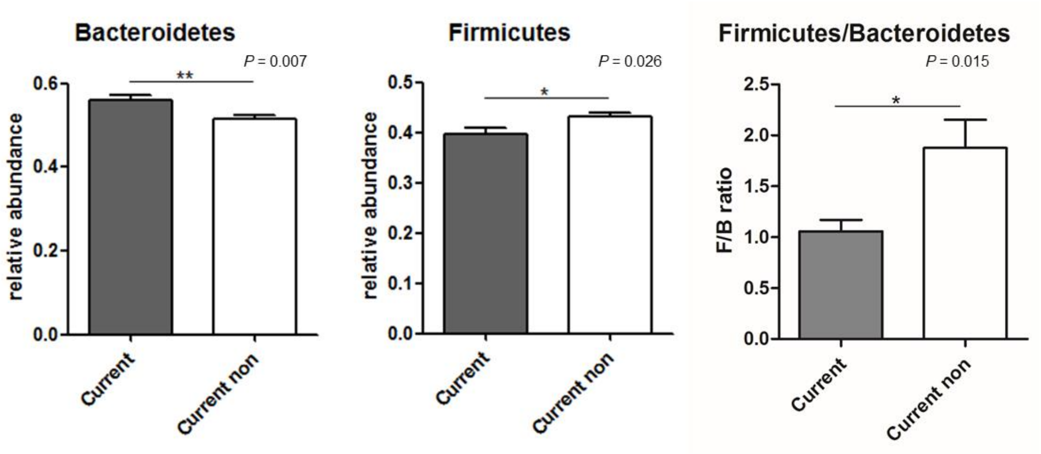

Table S1 Results of analysis of composition microbiomes

Level	Taxonomic assignment	W
**Never vs. current**		
**Class**	None	
**Order**	k__Bacteria;p__Bacteroidetes;c__Bacteroidia;o__Bacteroidales (increased in current smoker) *	1
	k__Bacteria;p__Fusobacteria;c__Fusobacteriia;o__Fusobacteriales (decreased in current smoker)	1
	k__Bacteria;p__Proteobacteria;c__Gammaproteobacteria;o__Enterobacteriales (decreased in current smoker)	2
**Genus**	k__Bacteria;p__Bacteroidetes;c__Bacteroidia;o__Bacteroidales;f__Bacteroidaceae;g__Bacteroides (increased in current smoker) *	3
	k__Bacteria;p__Bacteroidetes;c__Bacteroidia;o__Bacteroidales;f__Porphyromonadaceae;g__Parabacteroides (increased in current smoker) *	1
	k__Bacteria;p__Firmicutes;c__Bacilli;o__Lactobacillales;f__Lactobacillaceae;g__Lactobacillus (increased in current smoker) *	7
	k__Bacteria;p__Firmicutes;c__Clostridia;o__Clostridiales;f__Clostridiales_Incertae_Sedis_XII;g__Acidaminobacter (decreased in current smoker)	1
	k__Bacteria;p__Firmicutes;c__Clostridia;o__Clostridiales;f__Gracilibacteraceae;g__Gracilibacter (decreased in current smoker)	1
	k__Bacteria;p__Firmicutes;c__Clostridia;o__Clostridiales;f__Lachnospiraceae;g__Howardella (decreased in current smoker)	1
	k__Bacteria;p__Firmicutes;c__Clostridia;o__Clostridiales;f__Lachnospiraceae;g__Lachnospiracea_incertae_sedis (increased in current smoker) *	1
	k__Bacteria;p__Firmicutes;c__Clostridia;o__Clostridiales;f__Lachnospiraceae;g__Pseudobutyrivibrio (decreased in current smoker)	2
	k__Bacteria;p__Firmicutes;c__Clostridia;o__Clostridiales;f__Lachnospiraceae;g__Roseburia (increased in current smoker) *	5
	k__Bacteria;p__Firmicutes;c__Clostridia;o__Clostridiales;f__Peptococcaceae;g__Desulfotomaculum (decreased in current smoker)	4
	k__Bacteria;p__Firmicutes;c__Clostridia;o__Clostridiales;f__Ruminococcaceae;g__Acetanaerobacterium (decreased in current smoker)	12
	k__Bacteria;p__Firmicutes;c__Clostridia;o__Clostridiales;f__Ruminococcaceae;g__Acetivibrio (decreased in current smoker)	1
	k__Bacteria;p__Firmicutes;c__Clostridia;o__Clostridiales;f__Ruminococcaceae;g__Clostridium_III (decreased in current smoker)	3

	k__Bacteria;p__Firmicutes;c__Clostridia;o__Clostridiales;f__Ruminococcaceae;g__Ethanoligenens (decreased in current smoker)	1
	k__Bacteria;p__Firmicutes;c__Clostridia;o__Clostridiales;f__Ruminococcaceae;g__Sporobacter (decreased in current smoker)	1
	k__Bacteria;p__Firmicutes;c__Negativicutes;o__Selenomonadales;f__Acidaminococcaceae;g__Acidaminococcus (decreased in current smoker)	1
	k__Bacteria;p__Fusobacteria;c__Fusobacteriia;o__Fusobacteriales;f__Fusobacteriaceae;g__Fusobacterium (decreased in current smoker)	1
	k__Bacteria;p__Proteobacteria;c__Betaproteobacteria;o__Burkholderiales;f__Comamonadaceae;g__Schlegelella (decreased in current smoker)	1
	k__Bacteria;p__Proteobacteria;c__Gammaproteobacteria;o__Enterobacteriales;f__Enterobacteriaceae;g__Citrobacter (decreased in current smoker)	1
	k__Bacteria;p__Tenericutes;c__Mollicutes;o__Anaeroplasmatales;f__Anaeroplasmataceae;g__Asteroleplasma (decreased in current smoker)	1
**Former vs. current**		
**Class**	None	
**Order**	None	
**Genus**	None	
**Never vs. former**		
**Class**	None	
**Order**	None	
**Genus**	None	
**Current non-smoker (never + former) vs. current**		
**Phylum**	k__Bacteria;p__Bacteroidetes (increased in current smoker) *	4
**Class**	k__Bacteria;p__Fusobacteria;c__Fusobacteriia (decreased in current smoker)	1
	k__Bacteria;p__Proteobacteria;c__Gammaproteobacteria (decreased in current smoker)	2
**Order**	k__Bacteria;p__Bacteroidetes;c__Bacteroidia;o__Bacteroidales (increased in current smoker) *	1
	k__Bacteria;p__Fusobacteria;c__Fusobacteriia;o__Fusobacteriales (decreased in current smoker)	1

	k__Bacteria;p__Proteobacteria;c__Gammaproteobacteria;o__Enterobacteriales (decreased in current smoker)	2
**Family**	k__Bacteria;p__Bacteroidetes;c__Bacteroidia;o__Bacteroidales;f__Bacteroidaceae (increased in current smoker) *	5
	k__Bacteria;p__Bacteroidetes;c__Bacteroidia;o__Bacteroidales;f__Rikenellaceae (decreased in current smoker)	1
	k__Bacteria;p__Firmicutes;c__Clostridia;o__Clostridiales;f__Clostridiales_Incertae_Sedis_XII (decreased in current smoker)	1
	k__Bacteria;p__Firmicutes;c__Clostridia;o__Clostridiales;f__Gracilibacteraceae (decreased in current smoker)	1
	k__Bacteria;p__Fusobacteria;c__Fusobacteriia;o__Fusobacteriales;f__Fusobacteriaceae (decreased in current smoker)	1
	k__Bacteria;p__Proteobacteria;c__Gammaproteobacteria;o__Enterobacteriales;f__Enterobacteriaceae (decreased in current smoker)	2
**Genus**	k__Bacteria;p__Bacteroidetes;c__Bacteroidia;o__Bacteroidales;f__Bacteroidaceae;g__Bacteroides (increased in current smoker) *	3
	k__Bacteria;p__Firmicutes;c__Bacilli;o__Lactobacillales;f__Lactobacillaceae;g__Lactobacillus (increased in current smoker) *	1
	k__Bacteria;p__Firmicutes;c__Clostridia;o__Clostridiales;f__Lachnospiraceae;g__Roseburia (increased in current smoker) *	1
	k__Bacteria;p__Firmicutes;c__Clostridia;o__Clostridiales;f__Ruminococcaceae;g__Acetanaerobacterium (decreased in current smoker)	10
	k__Bacteria;p__Firmicutes;c__Clostridia;o__Clostridiales;f__Ruminococcaceae;g__Acetivibrio (decreased in current smoker)	1
	k__Bacteria;p__Firmicutes;c__Clostridia;o__Clostridiales;f__Ruminococcaceae;g__Clostridium_IV (decreased in current smoker)	1
	k__Bacteria;p__Firmicutes;c__Clostridia;o__Clostridiales;f__Ruminococcaceae;g__Ethanoligenens (decreased in current smoker)	1
	k__Bacteria;p__Firmicutes;c__Negativicutes;o__Selenomonadales;f__Acidaminococcaceae;g__Acidaminococcus (decreased in current smoker)	1
	k__Bacteria;p__Fusobacteria;c__Fusobacteriia;o__Fusobacteriales;f__Fusobacteriaceae;g__Fusobacterium (decreased in current smoker)	1
	k__Bacteria;p__Proteobacteria;c__Betaproteobacteria;o__Burkholderiales;f__Comamonadaceae;g__Schlegelella (decreased in current smoker)	1
	k__Bacteria;p__Proteobacteria;c__Deltaproteobacteria;o__Desulfovibrionales;f__Desulfovibrionaceae;g__Bilophila (decreased in current smoker)	1
	k__Bacteria;p__Proteobacteria;c__Gammaproteobacteria;o__Enterobacteriales;f__Enterobacteriaceae;g__Citrobacter (decreased in current smoker)	1

k, kingdom; p, phylum; c, class; o, order; f, family; * Marking bacteria are increased in current smoker

Table S2. Baseline characteristic of study population between Current non-smoker and current smokers

	Current non-smoker (*N*=555)	current smoker (*N*=203)	*P*-value
Age, year	45.6±9.0	45.7±8.2	0.947
BMI, kg/m^2	24.6±2.8	24.8±3.1	0.580
Muscle mass, kg	52.7±5.6	53.2±6.0	0.304
Fat mass, kg	17.2±5.6	17.2±5.7	0.879
Creatinine, mg/dL	1.00±0.12	0.97±0.13	0.005
eGFR, MDRD, ml/min/1.73m^2	87.9±12.4	91.5±14.6	0.002
Iron, µg/dL	127.0±38.5	127.9±38.7	0.813
Ferritin, ng/mL	210.4±123.1	224.8±164.4	0.199
C-reactive protein, mg/dL	0.11±0.19	0.12±0.19	0489
Comorbidities			
Diabetes mellitus	38 (6.8)	15 (7.4)	0.872
Hypertension	113 (20.4)	36 (17.7)	0.470
COPD	22 (4)	15 (7.4)	0.202
Liver disease*	104 (18.7)	33 (16.3)	0.457
Dyslipidemia	109 (19.6)	41 (20.2)	0.918
Kidney disease†	24 (4.3)	7 (3.4)	0.683
Spirometry			
FVC, % predicted	89.4±9.1	90.2±9.5	0.293
FEV_1, % predicted	89.6±9.3	88.7±10.7	0.583
FEV_1/FVC (%)	80.5±5.9	79.3±6.9	0.015
Nutrition			
Total energy, kcal/day	1453.0±623.0	1523.4±634.9	0.236
Total protein, g/day	50.0±25.6	53.1±25.4	0.199
Total fat, g/day	29.0±19.5	30.8±19.3	0.328
Total carbohydrate, g/day	243.8±103.5	253.7±111.3	0.326
Total calcium mg/day	301.4±194.5	306.6±186.5	0.772
Total phosphorus, mg/day	721.0±342.3	759.1±344.2	0.242
Total vitamin A, ug/day	310.6±213.9	344.8±230.4	0.099
Total sodium, mg/day	1596.5±1010.1	1809.8±1093.8	0.030
Vitamin B1, mg/day	0.85±0.45	0.91±0.46	0.115
Vitamin C, mg/day	65.1±53.1	66.7±47.2	0.739
Folate, mg/day	143.6±92.8	152.8±93.5	0.297
Retinol, ug/day	71.4±58.5	72.9±60.6	0.786
Fiber, g/day	3.6±2.0	3.8±2.1	0.319
Cholesterol, mg/day	171.6±145.8	182.6±151.5	0.431

Values represented as mean ± standard deviation or N (%); BMI, body mass index; COPD, chronic obstructive pulmonary disease; FVC, forced vital capacity; FEV_1, forced expiratory volume in one second; eGFR, estimated glomerular filtration rate; MDRD, Modification of Diet in Renal Disease; *Liver disease including hepatitis B, hepatitis C, liver cirrhosis, fatty liver; †Kidney disease including chronic kidney disease, ureter stone, benign prostate hypertrophy
